# Supplementation of Methyl-Donor Nutrients to a High-Fat, High-Sucrose Diet during Pregnancy and Lactation Normalizes Circulating 25-Dihydroxycholecalciferol Levels and Alleviates Inflammation in Offspring

**DOI:** 10.3390/metabo12121252

**Published:** 2022-12-12

**Authors:** Chin May Teoh, Analynn Cooper, Karisa M. Renteria, Michelle Lane, Jie Zhu, Gar Yee Koh

**Affiliations:** Nutrition and Foods Program, School of Family and Consumer Sciences, Texas State University, San Marcos, TX 78666, USA

**Keywords:** vitamin D, obesogenic diet, methyl-donor nutrients, inflammation, maternal-fetal nutrition, epigenetic, DNA methylation

## Abstract

A Western-style diet that is high in fat and sucrose has been shown to alter DNA methylation and epigenetically modify genes related to health risk in offspring. Here, we investigated the effect of a methyl-donor nutrient (MS) supplemented to a high-fat, high-sucrose (HFS) diet during pregnancy and lactation on vitamin D (VD) status and inflammatory response in offspring. After mating, 10-week-old female Sprague-Dawley (SD) rats (*n* = 10/group) were randomly assigned to one of the four dietary groups during pregnancy and lactation: (1) control diet (CON), (2) CON with MS (CON-MS), (3) HFS, and (4) HFS with MS (HFS-MS). Weanling offspring (three weeks old) were euthanized and sacrificed (*n* = 8–10/sex/group). The remaining offspring (*n* = 10/sex/group) were randomly assigned to either a CON or an HFS diet for 12 weeks and sacrificed at 15 weeks of age. Our results indicated that prenatal MS supplementation, but not postnatal diet, restored low vitamin D status and suppressed elevation of proinflammatory cytokine induced by maternal HFS in the offspring. Furthermore, both prenatal and postnatal diets modulated the abundance of *Lactobacillus* spp. and *Bacteroides* spp. in the offspring, a shift that was independent of vitamin D status. Collectively, our data support a role for MS in restoring the perturbation of VD status and normalizing maternal HFS-induced inflammation in the offspring. Further investigation is warranted to elucidate the methylation status of VD metabolism-related pathways in the offspring, as well as the immunomodulatory role of vitamin D during the progression of obesity.

## 1. Introduction

In a recent 20-year analysis, the prevalence of overweight children and childhood obesity had increased from 2% to 5% between 1990 and 2018 [1]. An association between maternal dietary pattern with growth trajectories and risks of childhood obesity has been demonstrated lately, whereby a higher intake of fast food, characterized by high saturated fat and added sugar, or commonly known as the Western diet, contributed to a higher risk of children being obese by the age of four [2]. Consumption of a high-fat diet (60% kcal from fat) during pregnancy and lactation has been linked to increased inflammation and metabolic dysfunctions in vivo [3,4]. In addition, higher maternal healthy eating index (HEI) had sex-specific associations with phenotypic changes in inflammation and growth factor signaling [5].

Vitamin D (VD) presents naturally either in the form of cholecaclciferol (vitamin D_3_) from sunlight and animal sources or in ergocalciferol (vitamin D_2_) from plant sources. VD is absorbed in the form of D_3_ and binds to vitamin D binding protein to be transported in blood circulation to the liver. In the liver, vitamin D_3_ is converted into 25-hydroxyvitamin D (25D), which is then further hydroxylated into its active form, 1α,25-dihydroxy vitamin D (1,25D) by 1α-hydroxylase enzyme (encoded by *Cyp27b1* gene) in the kidneys. Homeostasis of 1,25D levels can be maintained by the expression of VD receptor (VDR) through upregulation or downregulation of the 1α-hydroxylase enzyme and the 24-hydroxylase enzyme (encoded by *Cyp24a1* gene), which catabolizes 1,25D into 1-24,25-trihydroxyvitamin D3 to be excreted out of the body, respectively. Low VD status and changes in genes related to VD metabolism are commonly observed among offspring born to dams on a Western dietary pattern [6,7]. Moreover, suboptimal VD status (serum 25D < 30 ng/mL) is strongly correlated with incidences of inflammatory bowel disease, cancers, autoimmune diseases, and immune dysfunction [8,9,10]. Furthermore, VD supplementation has been shown to regulate the gut microbiome and protect against intestinal inflammation [11,12]. Recent studies have revealed that establishment of microbiota in the prenatal gut via in utero transfer may play a role in predisposition to diseases in later life [13,14]. However, the impact of these early gut microbiota on vitamin D metabolism is not widely investigated. In fact, study has shown that VDR knockout mice exhibited a lower abundance of *Lactobacillus* spp. and an increased abundance of *Bacteoroides* spp. [15]. Moreover, members from these bacteria genera foster the growth of butyrate-producing bacteria, such as *Eubacterium* spp. [16,17], to suppress inflammation, and were associated with energy metabolism [18,19,20]. These may suggest a potential anti-inflammatory role of these bacteria mediated by VD signaling. Another mechanism underlying the immunomodulatory role of VD could be mediated by the production of antimicrobial peptides, such as cathelicidin. Gene expression of cathelicidin is directly regulated by VDR upon binding to 1,25D, a common ligand for VDR [21]. In septic obese mice, vitamin D_3_ has been shown to promote cathelicidin secretion and suppress systemic inflammation [22,23]. Furthermore, emerging evidence has demonstrated the ability of cathelicidin in attenuating the activation toll-like receptor 4 (TLR4), and thus suppressing the downstream inflammatory response [24,25].

A recent European prospective cohort study reported a diet rich in methyl-donor nutrients, such as folate, vitamin B_12_, choline, methionine, and betaine, was associated with global methylation changes [26], which were linked to risks of cardiometabolic diseases among offspring [27]. Previously, an in vivo rat study showed that maternal methyl-donor nutrient deficiency downregulated the gene expression of *VDR* and reduced the bioavailability of active VD [28]. However, the role of methyl-donor nutrients in modulating methylation status of VD-related genes under obesogenic environment remain unclear. In this study, we hypothesized that methyl-donor nutrient supplementation (MS) during pregnancy and lactation could improve VD status and alleviate maternal high-fat, high-sucrose (HFS) diet-induced inflammation among offspring. We further examined the changes of *Lactobacillus* spp., *Bacteroides* spp., and *Clostridium coccoides/Eubacterium rectale* group and their associations with colonic VD signaling and inflammation. For the first time, we demonstrate that prenatal MS prevents the perturbation of VD homeostasis and suppresses the elevation of proinflammatory cytokine induced by obesogenic maternal diet among the offspring. Due to the potential role of VD in immune and metabolic functions, early life optimization of VD status may serve as an effective strategy for the prevention of metabolic diseases.

## 2. Materials and Methods

### 2.1. Animal Design

Eight-week-old Sprague–Dawley (SD) dams and sires were purchased from Charles River Laboratories (Wilmington, MA, USA) and pair-housed at 25 °C and 70% humidity with a 12-h light/dark cycle prior to mating. During the two-week acclimation period, all rats consumed the control (CON), AIN-93G diet (D10012G, Research Diets, New Brunswick, NJ, USA). During mating, one dam was housed with one sire for one week and confirmation of pregnancy was obtained from the first positive vaginal smear for spermatozoa. After breeding, female rats were randomly assigned to one of four dietary groups (*n* = 10/group): (1) CON, (2) CON with methyl-donor nutrient supplementation (CON-MS), (3) high-fat, high-sucrose diet (HFS, D2011906, Research Diets), and (4) HFS with methyl-donor nutrient supplementation (HFS-MS). CON and HFS diets contained adequate levels of methyl-donor nutrients for pregnancy and growth. The following methyl-donor nutrients were added based on a weight basis to the supplemented groups: vitamin B_12_ (1.5 mg/kg), folic acid (15 mg/kg), betaine (15 mg/kg), choline (11.2 mg/kg), L-methionine (7.5 mg/kg), and zinc (34.1 mg/kg). Dams were housed in pairs upon arrivals and throughout the first week of gestational period for a total of four weeks. Then, the dams were separated and housed individually for the last two weeks of gestation and throughout lactation period. All pups were weaned at three weeks of age. At weaning, a portion of the three-week-old pups (*n* = 8–10/sex/group) were euthanized and blood, cecal contents, and colonic mucosa were collected. The remaining pups consumed either the CON or HFS diet (nonirradiated diets) for the following 10 weeks (*n* = 10/sex/group). At 10 weeks postnatal, the adult pup diets were switched to irradiated diets, corresponding to their treatments, through sacrifice to prevent potential diet contamination that may confound microbiome analysis. All diets were consumed ad libitum. At 15 weeks of age, adult pups were euthanized and sacrificed to collect blood, cecal contents, and colonic mucosa. All samples were kept at −80 °C until analysis. All experimental procedures were approved by the Institutional Animal Care and Use Committee (IACUC) at Texas State University, protocol #7616.

### 2.2. Analysis of Serum 25D Concentrations

Serum was separated from blood via centrifugation at 1500× *g* for 15 min. Serum concentrations of 25D were measured with a commercially available ELISA kit (Crystal Chem, Elk Grove Village, IL, USA) according to manufacturer’s instructions.

### 2.3. Analysis of Proinflammatory Cytokines

Plasma was collected from blood after centrifugation at 1500× *g* for 15 min. Circulating levels of proinflammatory cytokine IL-1β were measured with a commercially available ELISA kit (Invitrogen, Waltham, MA, USA). All procedures and sample dilutions were performed based on the manufacturer’s instructions.

### 2.4. RNA Extraction and Real-Time PCR Analysis

Colons were excised and opened longitudinally, then rinsed with ice-cold phosphate-buffered saline. The mucosa was scraped from the colon by using a glass microscope slide and stored at −80 °C until analysis. For RNA extraction, the colonic mucosa was homogenized and the RNA was isolated by using Trizol^®^ solution (Invitrogen, Waltham, MA, USA). RNA concentrations were quantified by using a Nanodrop Lite (Thermo Fisher, Waltham, MA, USA) and single-strand cDNA was synthesized by using a SuperScript IV cDNA reverse transcription kit (Invitrogen, Waltham, MA, USA) as per the manufacturer’s instructions. Real-time PCR reactions were performed in duplicate by using PowerUp SYBR Green Detection reagents (Applied Biosystems, Waltham, MA) for detection of *Gapdh*, *Vdr*, cathelicidin, *Il6*, *Il1β*, and *Tlr4* with a QuanStudio-3 PCR machine (Applied Biosystems, Waltham, MA, USA). The primer sets specific for each target were presented in Table S1. Expression of each target gene was normalized against *Gapdh* and relative expression was determined as mean fold change in gene expression relative to weanling pups of CON dams or adult pups of CON dams fed a CON diet, respectively.

### 2.5. Microbiome Analysis

QIAamp^®^ DNA extraction kits (Qiagen, Hilden, Germany) were used to extract DNA from the cecal contents. Detection of the selected bacteria species, *Eubacterium rectale*, *Bacteroides* spp., and *Lactobacillus* spp. was conducted by using real-time PCR reaction as described above using 200 ng of DNA [29]. Supplementary Table S1 includes the primer pairs for each target species. Expression of these target bacteria species is normalized against *Eubacteria* and is presented as fold changes in gene expression relative to weanling pups of CON dams or adult pups of CON dams fed a CON diet., respectively.

### 2.6. Statistical Analysis

All statistical analyses were performed by using SigmaPlot version 14.5 (Palo Alto, CA, USA). Data from weanling pups were conducted by using one-way analysis of variance (ANOVA). Data from adult offspring were analyzed by using two-way ANOVA. All data were followed by Tukey’s post hoc analyses or Dunn’s multiple comparison for unequal groups. All data were normally distributed and correlations of colonic VDR mRNA expression and colonic proinflammatory markers mRNA expression were determined by Pearson’s correlation coefficient. For mRNA expression data, statistical analyses were conducted by using data from ∆Ct, and relative expression values are reported as indicated. *p*-values < 0.05 were considered significant. All data are presented as mean ± standard error, unless otherwise specified. The data presented in this study are pooled from both male and female pups, as no sex interaction was found.

## 3. Results

### 3.1. MS Supplementation during Pregnancy and Lactation Restores Perturbation of Vitamin D Status in Adult Offspring Induced by Maternal HFS Diet

**Figure 1:**
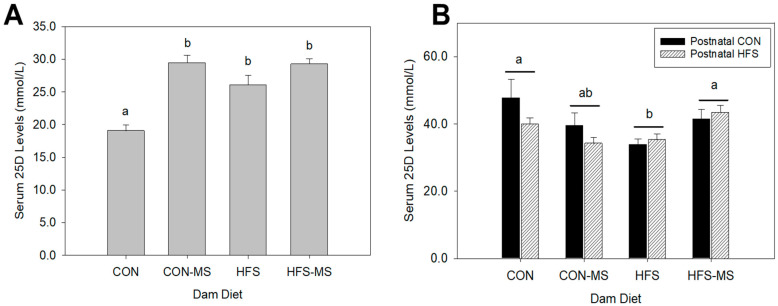
Circulating 25-hydroxycholecalciferol (25D) in weanling (**A**) and adult (**B**) offspring born to dams fed a control (CON) or high-fat, high sucrose (HFS) diet with or without methyl-donor nutrient supplementation (MS). Data are pooled from both sexes. Different letters indicate significant differences between maternal diets at *p* < 0.05. Data are expressed as mean ± SEM (*n* = 10/group). Postnatal CON, control postnatal diet; postnatal HFS, high-fat, high-sucrose postnatal diet.

At weaning, VD status, as indicated by circulating levels of 25-hydroxycholecalciferol (25D), was 25% higher (*p* < 0.01) in pups born to dams that consumed the HFS diet compared to pups born dams that consumed the CON diet. The 25D did not differ between pups whose dams consumed the CON, CON-MS, or HFS-MS diets (Figure 1A). To further determine if maternal VD status affects circulating levels of 25D, we tested the serum 25D levels in dams; however, we did not observe significant differences in circulating 25D levels in dams across all groups (Figure S1). Following 12 weeks of dietary interventions, adult pups born to HFS dams exhibited 20% less circulating 25D compared to adult pups born to CON dams regardless of the diet consumed by pups after weaning (*p* = 0.013) (Figure 1B). Moreover, MS supplementation to HFS diet, but not CON diet, during pregnancy and lactation significantly enhanced VD status in their offspring by 25% (*p* = 0.043), resulting in levels similar to those observed in pups whose dams consumed the CON diet (Figure 1B). Postnatal diets (postnatal CON vs. postnatal HFS) did not affect VD status of adult pups (15 weeks of age) within each dam diet (Figure 1B).

### 3.2. MS Supplementation during Pregnancy and Lactation Attenuates Maternal HFS-Induced Inflammation in Adult Pups

**Figure 2:**
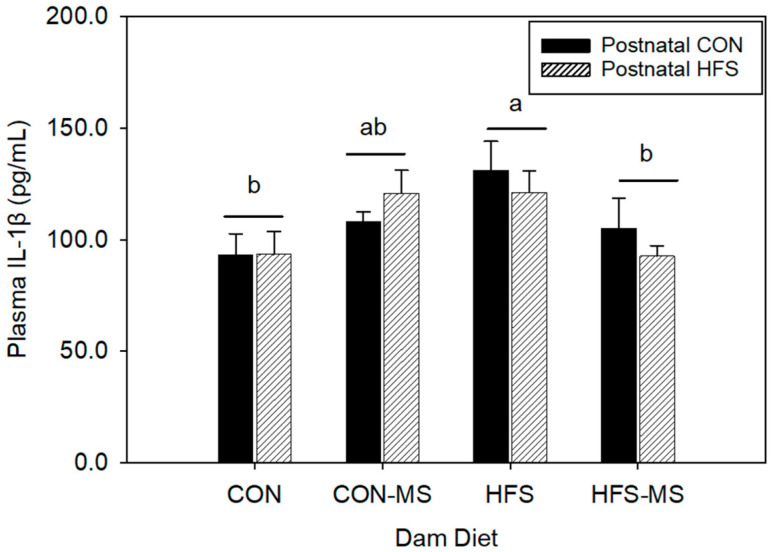
Plasma levels of IL-1β in adult offspring born to dams fed a control (CON) or high-fat, high sucrose (HFS) diet with or without methyl-donor nutrient supplementation (MS). All data are pooled from both sexes. Different letters indicate significant differences between maternal diets at *p* < 0.05. Data are expressed as mean ± SEM (*n* = 10/group). Postnatal CON, control postnatal diet; postnatal HFS, high-fat, high-sucrose postnatal diet.

To further evaluate the inflammatory status in the adult pups, we determined the circulating levels of proinflammatory cytokines, IL-1β and IL6, by using commercially available ELISA kits. Plasma IL-1β levels in adult pups born to dams that consumed the HFS diet were higher than those born to dams that consumed the CON diet (*p* = 0.01) regardless of the diet that the pups received after weaning (Figure 2). Compared to pups born to HFS dams, circulating IL-1β concentrations were 24% lower in pups of HFS-MS dams (*p* = 0.038). Circulating IL-1β concentrations did not differ between pups born to HFS-MS and CON dams. Similarly, no difference was observed between pups of CON dams and pups of CON-MS dams. Circulating IL-6 concentrations were also investigated; however, plasma IL-6 levels were too low to be detected in these pups.

### 3.3. Maternal Diets Differentially Altered Colonic Vitamin D Signaling in Pups at Weaning and Adulthood

**Figure 3:**
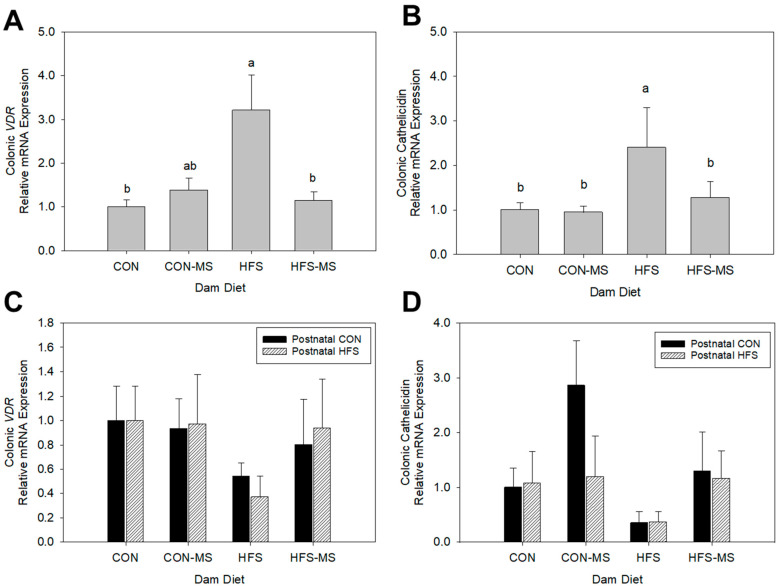
Relative mRNA expression of colonic *VDR* (**A**,**C**) and cathelicidin (**B**,**D**) in weanling (**A**,**B**) and adult (**C**,**D**) offspring born to dams fed control (CON) or high-fat, high-sucrose (HFS) diets with or without methyl-donor nutrient supplementation (MS). Data are normalized to GAPDH and expressed in relative to pups of CON dam (weanling) or pups of CON dams fed a p-CON diet (adult). All data are pooled from both sexes. Different letters indicate significant differences between maternal diets at *p* < 0.05. Data are expressed as mean ± SEM (*n* = 6–10/group). Postnatal CON, control postnatal diet; postnatal HFS, high-fat, high-sucrose postnatal diet.

At weaning, mRNA expression of colonic *VDR* (*p* < 0.01) and its downstream target, cathelicidin (*p* = 0.02), in pups born to dams that consumed the HFS diet were upregulated by threefold compared to pups born to CON dams (Figure 3A,B). When dams’ HFS diet was supplemented with MS throughout the gestation and lactation periods, mRNA expression of colonic *VDR* in their offspring was downregulated by threefold (*p* = 0.01), to levels similar to those observed in pups of dams consuming the CON diet. A similar trend was observed for cathelicidin (*p* = 0.02), when pups of HFS dams are compared to those of HFS dams (Figure 3A,B).

At 15 weeks of age, the mRNA expression of colonic *VDR* was numerically lower in pups born to HFS dams than in pups born to CON dams, but this effect failed to reach significance (*p* = 0.42) (Figure 3C,D). However, a lowering trend was observed with the mRNA expression of colonic cathelicidin in pups born to HFS dams compared to those of CON dams (*p* = 0.09). To determine if maternal or pup diets may have altered the synthesis of active VD (1,25-dihydroxycholecalciferol) in the colon, we further investigated the expression of the mRNA encoding the *Cyp27b1* enzyme. However, no difference was detected between all groups at either weaning or adulthood (Figure S2).

VD plays a critical role in immune regulation. Hence, we further explored the relationship between colonic VDR and these proinflammatory markers. Positive correlations were identified between the mRNA expressions of colonic *VDR* and *Il6*, as well as a trend for positive correlation with *Il1β* and *Tlr4* in three-week-old weanling pups (Table 1). Significant positive correlations continued throughout adulthood as observed in colonic mucosa from 15-week-old adult pups (Table 1). We did not detect any differences of the mRNA expressions of proinflammatory markers, including *Tlr4*, *Il1β*, and *Il6* (Figure S3), respectively.

### 3.4. Prenatal and Postnatal Diets Together Modulate the Abundance of Bacteroides spp. and Lactobacillus spp. in Adult Pups

**Figure 4:**
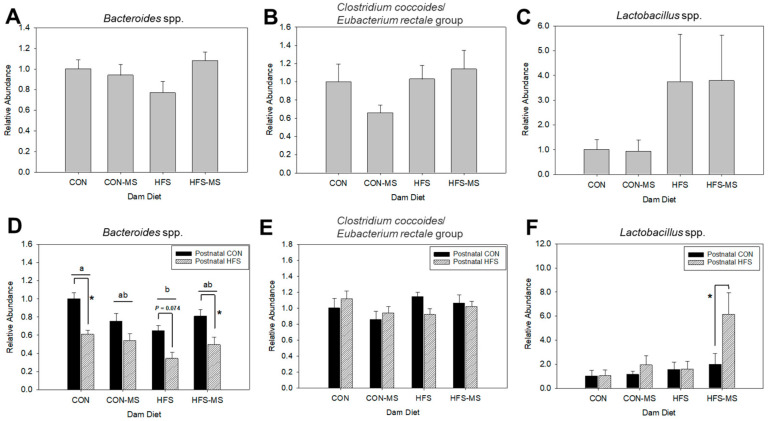
Relative abundance of *Bacteroides* spp. (**A**,**D**), *Clostridium coccoides/Eubacterium rectale* group (**B**,**E**), and *Lactobacillus* spp. (**C**,**F**) in weanling (**A**–**C**) and adult (**D**–**F**) offspring born to dams fed control (CON) or high-fat, high-sucrose (HFS) diets with or without methyl-donor nutrient supplementation (MS). Data are normalized to *Eubacteria* and expressed in relative to pups of CON dam (weanling) or pups of CON dams fed a postnatal CON diet (adult). All data are pooled from both sexes. Different letters indicate significant differences between dam diets at *p* < 0.05. * Indicates significant difference between pup’s diets at *p* < 0.05. Data are expressed as mean ± SEM (*n* = 6–10/group). Postnatal CON, control postnatal diet; Postnatal HFS, high-fat, high-sucrose postnatal diet.

To investigate if maternal MS supplementation influences early establishment of gut microbiota in their offspring, we evaluated the abundance of selected groups of gut microbiota in the offspring at weaning and 15 weeks of age, respectively (Figure 4). No changes in the *Bacteroides* spp., *Eubacterium rectale/Clostridium coccoides* group, and *Lactobacillus* spp. were observed in weanling pups (Figure 4A–C). At 15 weeks old, differential changes were observed in *Bacteroides* spp. and *Lactobacillus* spp., but not the *Eubacterium rectale/Clostridium coccoides* group. Specifically, the abundance of *Bacteroides* spp. was lower in offspring born to HFS dams, compared to offspring of CON dams (*p* = 0.01). The offspring microbiota examined were not impacted by maternal MS supplementation (Figure 4D–F). Postnatal HFS diet further suppressed the abundance of *Bacteroides* spp. among the offspring if their dams consumed the CON or HFS diets, but this effect did not reach significance in pups whose dams consumed CON-MS or HFS-MS diets (Figure 4E). In contrast, the abundance of *Lactobacillus* spp. was not affected by maternal diet with one exception. Specifically, the adult offspring born to HFS-MS dams exhibited a threefold increase in *Lactobacillus* spp. when fed a postnatal HFS diet compared to those fed a postnatal CON diet (*p* = 0.04) (Figure 4F).

## 4. Discussion

Our current results demonstrate that consumption of a HFS diet during pregnancy and lactation by dams adversely impacts offspring VD status, signaling and, consequently, inflammation. At weaning, the addition of MS to a HFS diet during pregnancy and lactation did not affect VD status, though colonic VD signaling was suppressed, when compared to pups born to nonsupplemented HFS dams and did not differ from pups of CON dams. At adulthood (i.e., 15 weeks of age), VD status among pups born to HFS-MS dams was improved compared to pups of HFS dams and did not differ from pups of CON dams. However, we did not observe significant changes with colonic VD signaling among adult pups. MS supplementation further attenuated maternal HFS-induced inflammation among adult pups as indicated by circulating IL-1β levels. Taken together, our current investigation suggests that prenatal diet, but not postnatal diet, may determine the phenotypical changes observed in this study, including VD and inflammatory status. Similarly, the abundance of selected groups of gut microbiota, specifically *Bacteroides* spp. and *Lactobacillus* spp., is dependent on maternal diet and the changes of these bacteria species can be exacerbated by postnatal diet. Because these genera of bacteria have shown to impact host energy metabolism, changes of these bacteria groups could be in response to the host energy shift resulting from HFS intake.

Methyl-donor nutrients play a crucial role in one-carbon metabolism, particularly in DNA methylation and epigenetic changes. Maternal high-fat dietary intake has shown to induce DNA methylation changes that contribute to glucose intolerance and inflammation among offspring [30,31,32,33]. There are conflicting results in clinical and preclinical studies regarding the association between intake of methyl-donor nutrients, such as folate, choline, and methionine and chronic disease risk [34,35]. These may be attributed to the fact that most studies focus solely on one methyl-donor nutrient and not the interactions among these nutrients. To date, limited information is available with regard to the synergistic effect of these nutrients as a dietary pattern. Our study utilized a combination of several methyl-donor nutrients with the goal of optimizing one-carbon metabolism and correct the epigenetic changes typically associated with high-fat high-sucrose intake, such as VD deficiency, impaired glucose tolerance, and chronic low-grade inflammation.

Studies on VD and its transgenerational effects in disease prevention and progression generally focused on VD deficiency or supplementation during pregnancy, lactation, or both [36,37]. We showed that maternal intake of fat and sucrose, particularly during gestational and lactation stage, could impact VD status in their offspring, and that it was independent from VD status among the dams. Assessment of VD status among offspring resulted in differential levels at weaning and at 15 weeks of age, respectively. Specifically, serum 25D levels were 50% higher in weanling pups of CON-MS dams compared to those born to CON dams; however, maternal MS supplementation did not affect VD status in weanling pups born to HFS dams, though compared to pups of CON dams, serum 25D levels in pups of HFS or HFS-MS dams were 1.5-fold higher. Purcell et al. previously demonstrated that higher lipid content was found in breast milk on postnatal day 21 from dams on a high-fat diet [38]. We speculated that HFS might cause an elevation of lipid levels in dams and, therefore, might enhance the solubilization of VD in breast milk in HFS-fed dams. Consequently, the 25D levels could be optimized in weanling pups of HFS dams due to increased solubility of VD in breast milk, regardless of prenatal MS supplementation. This is likely a transient effect during the lactation period because at 15 weeks of age lower levels of 25D were observed in pups born to HFS dams relative to pups born to CON dams. It is important to note that, though we did not observe an improvement in 25D status among pups born to HFS-MS dams at weaning compared to those born to HFS dams, serum 25D levels were enhanced in adult offspring (15 weeks of age) born to HFS-MS dams. We speculated that perturbation of VD status induced by maternal HFS diet may become prominent as the metabolism stabilized at adulthood, which may be mitigated by MS supplementation as evidenced in our study. Studies have shown that maternal VD status altered *VDR* gene expression and serum 25D levels among fetal and adult offspring [39,40]. However, we did not observe changes with maternal VD status in our study, suggesting that other factors, such as the maternal inflammatory status, could have contributed to the epigenetic modulation of VD-related signaling. The relationship between maternal inflammation status and impact on vitamin D status in offspring remains to be elucidated.

The functional role of VD beyond bone health is well recognized. In the past two decades, the role of VD in other phenomena, including cardiovascular health, autoimmune diseases, and various cancers, has been elucidated [41]. The immunomodulatory role of VD has further demonstrated in infectious diseases and metabolic diseases [42,43]. Low VD status was often observed in obese individuals and weight loss was associated with increased level of 25D as shown in clinical studies [44,45]. This is further supported by a meta-analysis showing that supplementation of cholecalciferol reduces BMI and waist circumferences among obese and overweight individuals, and so may exert a potential weight loss effect [46]. Hence, optimization of VD status is critical in preventing the development or progression of obesity, which may serve as a viable strategy to prevent morbidity or mortality associated with obesity. It is believed that greater risk for developing gastrointestinal diseases, such as colorectal cancer and IBD, among the obese could be driven by low-grade inflammation in the gut [4]. We hypothesized that optimization of VD status can attenuate the obesity-induced colonic inflammation through the activation of VDR in the gut. In support of this concept, we demonstrated a positive correlation between colonic *VDR* and other proinflammatory cytokines (i.e., *Tlr4*, *Il6*, and *Il1β*) in the gut, confirming an immunomodulatory role of VD signaling in the gut. Furthermore, cathelicidin has been shown to activate TLR4 signaling and modulate inflammatory status in vivo [47]. Though it is still unclear how MS mediated the restorative effect of VD status among offspring, folate and vitamin B_12_ deficiencies have been shown to suppress VDR protein levels in bone cells in vitro [28]. This may indicate a critical role of MS nutrients in mediating VD signaling and subsequent inflammatory responses among the offspring. It is interesting that the mRNA expression of colonic *VDR* and cathelicidin were upregulated in weanling offspring born to HFS dams; yet, at 15 weeks postnatal, mRNA expressions of colonic *VDR* and cathelicidin appear to be lower in pups born to HFS dams compared to those of CON dams. Because we observed an increased levels of circulating IL-1β in adult pups born to HFS dams compared to those of CON dams, the reduced expressions of colonic *VDR* and proinflammatory cytokines in these pups may suggest a compensatory mechanism to suppress inflammation induced by a maternal HFS diet among the adult offspring. This further led us to the assumption that activation of colonic VD signaling in the weanling pups could be a transient response to maternal HFS intake or inflammatory status as these pups were dependent on breast milk prior to weaning. Further investigations will be required to examine maternal inflammatory status and its correlation to colonic VD signaling among the weanling pups. It is also important to note that mRNA expression of colonic *VDR* is not dependent on serum 25D circulating levels. Because no changes in colonic 1α- hydroxylase (*Cyp27b1*) were observed, we postulated that regulation of VDR in the colon could be mediated by other factors, such as inflammatory cytokines.

The finding that cathelicidin is a direct target of *VDR* may explain the functional role of VD in innate immune system, to some extent mediated by the gut microbial community. Specifically, Villa et al. demonstrated that maternal vitamin D supplementation programmed colonic *Bacteroides* in male offspring that was associated with improved bone health [48]. However, in our study, we did not observe significant interactions between the selected groups of gut microbiota and VD signaling. Rather, the gut microbiota was impacted by prenatal and postnatal diets, respectively, especially at adulthood, and the abundance of *Bacteroides* spp. was lower in offspring on born to HFS dams compared to those of CON dams; furthermore, it was reduced in offspring fed a HFS diet. Therefore, it is still unclear if the changes in VD signaling and inflammatory status are directly regulated by gut microbiota at this point. It is important to note that most studies that demonstrated VD-mediated alteration in the gut microbiome utilized VD-deficient, VD-supplemented, or *VDR* knockout models [8,9,48,49,50]. It is possible that the VD levels in the offspring were not sufficient to exert changes in the gut microbiota as all rats (both dams and offspring) were provided vitamin D-sufficient diets.

The selected gut microbiota in our study also represents various groups of gut bacteria that exert a role in host energy metabolism [18,19,20]. A study by Chu et al. revealed that exposure to a maternal high-fat diet during pregnancy persistently reduced the abundance of *Bacteroides* spp. in the infant gut [51]. Consistent with this finding, we observed a drastic change in *Bacteroides* spp. in 15-week-old pups due to postnatal diets, though we did not observe significant difference among weanling pups. It is possible that the lower gut microbial diversity and abundance at weaning attributed to the lack of changes in bacterial abundance. It is widely acknowledged that reduced levels of *Bacteroides* spp. are associated with obesogenic diet [19,51,52]. Our study confirmed these findings and demonstrated that consumption of a postnatal HFS diet suppressed the abundance of *Bacteroides* spp. Interestingly, this effect was more pronounced when the adult offspring were born to dams that also consumed the HFS diet compared to those born to dams that consumed the CON diet, suggesting both maternal and postnatal effects on *Bacteroides* spp. However, maternal MS supplementation to either a CON or HFS diet did not affect the abundance of *Bacteroides* spp. among their adult offspring. The observation in the *Bacteroides* spp. is in contrast with the abundance of *Lactobacillus* spp., in which it did not differ between adult offspring born to CON or HFS dams. However, MS supplementation to HFS diet during pregnancy and lactation led to an increased abundance of *Lactobacillus* spp. in adult offspring, specifically in those fed a HFS diet postnatally. Schaible et al. reported that maternal methyl-donor nutrients modified the risk of colitis in offspring that was consistent with prolonged microbiome shift [53]. Our study further justifies the potential of MS in programming gut microbiota among offspring. However, the microbiota programming by MS is rather selective, as we did not observe a consistent change with both the abundance of *Lactobacillus* spp. and *Bacteroides* spp. among the offspring. Further investigation is warranted to determine if supplementation of MS during pregnancy and lactation has a direct effect on the early gut microbiome establishment among the offspring or whether maternal MS intake indirectly modulates epigenetic changes relevant to energy metabolism among the offspring, hence altering substrates’ availability for the selected gut microbiota leading to a shift in gut microbial composition.

## 5. Conclusions

A maternal Western diet, characterized by high fat and high sugar intake, increases health risks among offspring, including dysregulated lipid metabolism, higher inflammation status, and low vitamin D status, possibly via epigenetic modulations. Our study supports the use of maternal MS to a HFS diet for the maintenance of VD signaling and regulation of inflammation among the offspring. Most importantly, our data suggest that vitamin D status and inflammation in the offspring are mainly driven by prenatal diet. These may further explain the discrepancies regarding the efficacy of vitamin D supplementation for the prevention of chronic diseases [54,55]. Though we did not observe a direct correlation between VD status and the selected gut microbiota, our study demonstrated that both pre- and postnatal diets independently affect the abundance of *Bacteroides* spp. and *Lactobacillus* spp. The mechanism by which maternal HFS and MS intake modulates the selected gut bacteria in their offspring remains to be elucidated. It is well recognized that vitamin D insufficiency is a public health concern as it enhances risk for metabolic comorbidities. Hence, our findings shed new light for metabolic disease prevention by optimizing VD status through prenatal dietary modifications.

## Figures and Tables

**Table 1:** Pearson’s correlations between mRNA expression of *VDR* and proinflammatory markers in weanling and adult offspring colon mucosa.

Weanling Pups (3-Weeks-Old)		
mRNA species	Pearson’s correlation coefficient (r) with colonic VDR	*p*-value
*Il1β*	0.265	0.07
*Il6*	0.405	<0.01
*Tlr4*	0.273	0.06
**Adult Pups (15-Weeks-Old)**		
mRNA species	Pearson’s correlation coefficient (r) with colonic VDR	*p*-value
*Il1β*	0.323	<0.01
*Il6*	0.294	<0.01
*Tlr4*	0.419	<0.01

All data are pooled from both sexes where *n* = 10/group.

## Data Availability

The data presented in this study are available in article and supplementary material.

## Supplementary Materials

The following supporting information can be downloaded at: https://www.mdpi.com/article/10.3390/metabo12121252/s1, Figure S1: Circulating levels of 25-hydroxycholecalciferol (25D) in dams fed a control (CON) or high-fat, high-sucrose (HFS) diet supplemented with or without methyl-donor nutrients (MS); Figure S2: Relative mRNA expression of *Cyp27b1* in the colon of weanling (A) and adult pups (B) born to dams fed a control (CON) or high-fat, high-sucrose (HFS) diet with or without methyl-donor nutrients supplementation (MS). Figure S3: Relative mRNA expression of *Il-1b* (A,D), *IL6* (B,E), and *TLR4* (C,F) in the colon of weanling (A–C) and adult pups (D–F) born to dams fed a control (CON) or high-fat, high-sucrose (HFS) diet with or without methyl-donor nutrients supplementation (MS). Table S1: List of primers set for RT-PCR examined in this study.
