# Supplementary material for: Supplementation of Methyl-Donor Nutrients to a High-Fat, High-Sucrose Diet during Pregnancy and Lactation Normalizes Circulating 25-Dihydroxycholecalciferol Levels and Alleviates Inflammation in Offspring
Source: Metabolites. 2022 Dec 12;12(12):1252. doi: 10.3390/metabo12121252 (PMC9783000; doi:10.3390/metabo12121252)
Supplement: Supplementary file 1 [file metabolites-12-01252-s001.zip › metabolites-2082971-supplementary.pdf]

metabolites

MDPI

Supplemental Figures and Tables

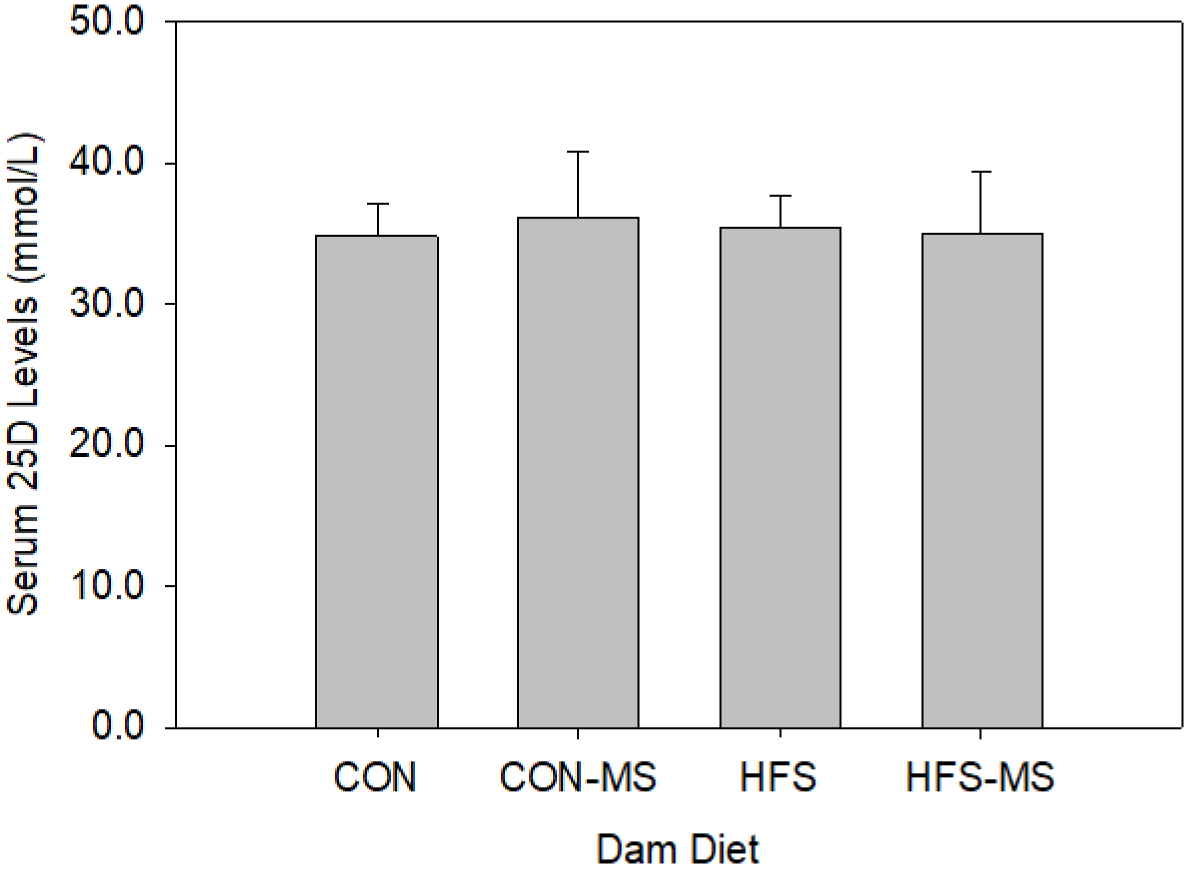

Figure S1. Circulating levels of 25-hydroxycholecalciferol (25D) in dams fed a control (CON) or high-fat high sucrose (HFS) diet supplemented with methyl-donor nutrients (MS). Data are expressed as mean ± SEM (n = 4/group)

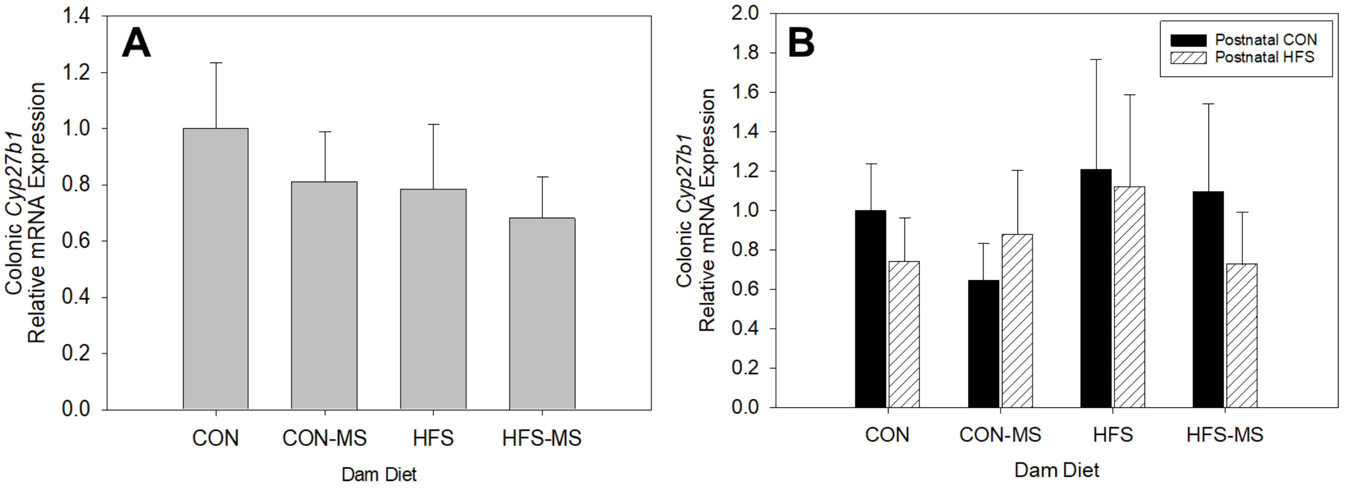

Figure S2. Relative mRNA expression of *Cyp27b1* in the colon of weanling (A) and adult pups (B) born to dams fed a control (CON) or high-fat high sucrose (HFS) diet with or without methyl-donor nutrients supplementation (MS). Data are normalized to GAPDH and expressed in relative to pups of CON dam (weanling) or pups of CON dams fed a postanal CON diet (adult). All data are pooled from both genders. Different letters indicate statistical difference maternal diets at $p < 0.05$. Data are expressed as mean ± SEM ($n = 6 – 10$/group). Postnatal CON, control postnatal diet; Postnatal HFS, high-fat high-sucrose postnatal diet

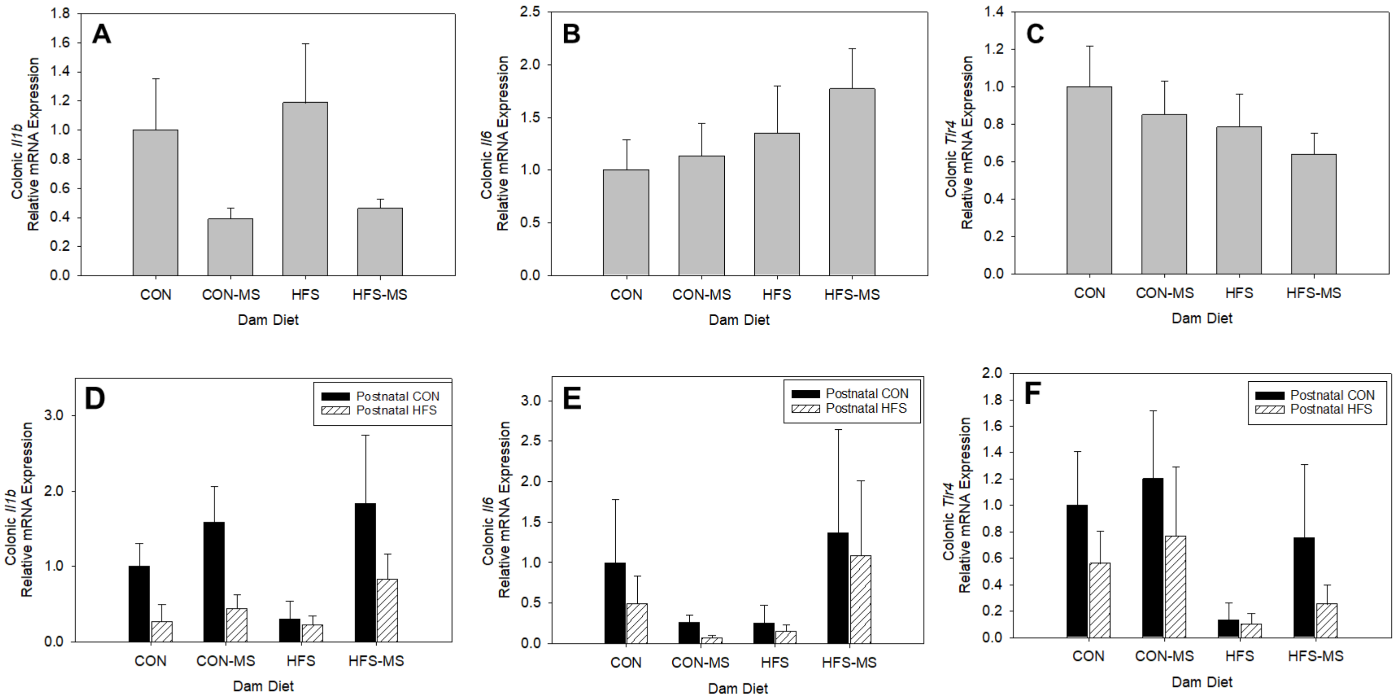

Figure S3. Relative mRNA expression of *Il1b* (A, D), *IL6* (B, E), *and TLR4* (C, F) in the colon of weanling (A, B, C) and adult pups (D, E, F) born to dams fed a control (CON) or high-fat high sucrose (HFS) diet with or without methyl-donor nutrients supplementation (MS). Data are normalized to GAPDH and expressed in relative to pups of CON dam (weanling) or pups of CON dams fed a postnatal CON diet (adult). All data are pooled from both genders. Different letters indicate statistical difference maternal diets at $p < 0.05$. Data are expressed as mean ± SEM (n = 6 – 10/group). Postnatal CON, control postnatal diet; Postnatal HFS, high-fat high-sucrose postnatal diet

Table S1: List of primers set for RT-PCR examined in this study.

Primer	Forward (5′ - 3′)	Reverse (5′ - 3′)
Gapdh	*GCA CAG TCA AGG CTG AGA AT*	*TGA AGA CGC CAG TAG ACT CC*
Vdr	*CCA TTC AGG ACC GCC TAT CC*	*GTC GGC CAG TTT CTG GAT CA*
Cyp27b1	*AAA GGT GTC TGT CCA GTC CA*	*CTC ATA GAG TGC CCA GGA GA*
Cathelicidin	*GGG TTG CCT CTA GCC GTT T*	*TGA AGT CAT CCA CAG CAG CAC GG*
Il1β	*GCA CAG TTC CCC AAC TGG TA*	*ACA CGG GTT CCA TGG TGA AG*
Il6	*GTT GCC TTC TTG GGA CTG ATG*	*ATA CTG GTC TGT TGT GGG TGG T*
Tlr4	*ACA GGG CAC AAG GAA GTA GC*	*GTT CTC ACT GGG CCT TAG CC*
Eubacteria (Universal)	*ACT CCT ACG GGA GGC AGC AGT*	*ATT ACC GCG GCT GCT GGC*
Bacteroides spp.	*GGT TCT GAG AGG AGG TCC C*	*CTG CCT CCC GTA GGA GT*
E. rectale/ C. coccoides Group	*ACT CCT ACG GGA GGC AGC*	*GCT TCT TAG TCA GGT ACC GTC A*
Lactobacillus spp.	*AGC AGT AGG GAA TCT TCC*	*CAC CGC TAC ACA TGG A*
